# Genomic Breed Composition of Selection Signatures in Brangus Beef Cattle

**DOI:** 10.3389/fgene.2020.00710

**Published:** 2020-07-10

**Authors:** Tiago do Prado Paim, El Hamidi A. Hay, Carrie Wilson, Milt G. Thomas, Larry A. Kuehn, Samuel R. Paiva, Concepta McManus, Harvey Blackburn

**Affiliations:** ^1^Instituto Federal de Educação, Ciência e Tecnologia Goiano, Iporá, Brazil; ^2^Universidade de Brasília, Brasília, Brazil; ^3^Fort Keogh Livestock and Range Research Laboratory, Agricultural Research Service, United States Department of Agriculture, Miles City, MO, United States; ^4^National Animal Germplasm Program, National Laboratory for Genetic Resources Preservation, Agricultural Research Service, United States Department of Agriculture, Fort Collins, CO, United States; ^5^Department of Animal Sciences, Colorado State University, Fort Collins, CO, United States; ^6^United States Meat Animal Research Center, Agricultural Research Service, United States Department of Agriculture, Clay Center, NE, United States; ^7^Embrapa Recursos Genéticos e Biotecnologia, Brasília, Brazil

**Keywords:** composite breeds, crossbreeding, local ancestry, *Bos taurus*, *Bos indicus*

## Abstract

Cattle breeding routinely uses crossbreeding between subspecies (*Bos taurus taurus* and *Bos taurus indicus*) to form composite breeds, such as Brangus. These composite breeds provide an opportunity to identify recent selection signatures formed in the new population and evaluate the genomic composition of these regions of the genome. Using high-density genotyping, we first identified runs of homozygosity (ROH) and calculated genomic inbreeding. Then, we evaluated the genomic composition of the regions identified as selected (selective sweeps) using a chromosome painting approach. The genomic inbreeding increased at approximately 1% per generation after composite breed formation, showing the need of inbreeding control even in composite breeds. Three selected regions in Brangus were also identified as Angus selection signatures. Two regions (chromosomes 14 and 21) were identified as signatures of selection in Brangus and both founder breeds. Five of the 10 homozygous regions in Brangus were predominantly Angus in origin (probability >80%), and the other five regions had a mixed origin but always with Brahman contributing less than 50%. Therefore, genetic events, such as drift, selection, and complementarity, are likely shaping the genetic composition of founder breeds in specific genomic regions. Such findings highlight a variety of opportunities to better control the selection process and explore heterosis and complementarity at the genomic level in composite breeds.

## Introduction

Breeding methods that exploit heterosis are common in livestock production. In cattle, the challenge for adopting terminal crossbreeding systems is consistent genetic composition of replacement heifers from the maternal breed (Lightner and Williams, 2018). Composite breeds (also referred to as synthetic breeds) allow for consistency in heterosis retention and heifer replacement. Brangus, developed in the United States, are an example of a composite breed, defined as 62.5% Angus and 37.5% Brahman (International Brangus Breeders Association^1^). The breed represents the complementarity between the tropically adapted *Bos taurus indicus* and the temperate high-valued carcass of *Bos taurus taurus* cattle (Gregory and Cundiff, 1980; Buzanskas et al., 2017). Brangus registration by the International Brangus Breeders Association started in 1949.

After a composite breed is formed, a genetic improvement program can be applied, selecting animals across generations based on expected progeny differences (EPD) for specific traits. The United States Brangus breeder association (IBBA) has developed EPDs for birth weight, weaning weight, yearling weight, milk production, total maternal, calving ease direct, calving ease maternal, scrotal circumference, ribeye area, and intramuscular fat (International Brangus Breeders Association;^1^). Therefore, artificial selection pressure, at varying levels of intensity, would have been employed on these traits. In this process, the inbreeding level can increase due the selection of few parents, especially bulls, and, consequently, decreasing heterosis. In this scenario, genomic selection signatures may arise after composite formation (Goszczynski et al., 2017). These genomic regions with selective sweeps may have different genomic breed composition than expected due to selective advantages of genes coming from one of the founder breeds.

The evaluation of selection signatures and genomic breed composition in composite breeds can contribute to a better understanding of the genetic effects associated with traits under selection and the inheritance of loci in crossbreeding systems (Grigoletto et al., 2019). Concerning the dynamics of composite breed development (Paim et al., 2020), we can gain new insights for crossbreeding systems based on a genomic perspective. The aim of this work was to expand our knowledge of composite breed genomics by identifying genomic inbreeding and selection signatures in Brangus. Further, we aimed to evaluate the genomic breed composition of these selected regions, identifying differential founder (Angus or Brahman) contributions to that region.

## Materials and Methods

### Animals

High-density SNP data (777,962 SNP, BovineHD Beadchip, Illumina, San Diego, CA, United States) from 68 Brahman, 95 Angus, and 59 prominent Brangus sires born from 1970 to 2010 were evaluated in total. Of the animals genotyped, 36 Brahman and 20 Brangus samples were acquired from the National Animal Germplasm Program’s (NAGP-ARS-USDA) gene bank (Fort Collins, CO, United States). The other samples were genotyped by the USMARC research center (ARS-USDA, Clay Center, NE, United States).

The Brangus pedigree, provided by the IBBA, consists of 1,152,050 individual animal records from which the genetic relationship coefficients were computed. The coefficient of genetic relationship was used to cluster the current Brangus population using Ward’s method in *proc cluster* of SAS University Edition (Copyright^©^ 2012–2018, SAS Institute Inc., Cary, NC, United States). Brangus were grouped into 17 clusters. The Brangus animals sampled for genotyping represented all 17 clusters. Sampled Brangus bulls were born in 12 states in the southern United States from 1970 to 2010, and these bulls had 43,393 progeny recorded by the IBBA.

### Pedigree Evaluation

The pedigree file was evaluated using the optiSel package (Wellmann, 2017) in R 3.4.2 software (R Core Team, 2017). The Angus, Brahman, and crossbred animals (with pedigree breed composition other than the 5/8 Angus, 3/8 Brangus) were considered as ancestors, totaling 75,449 ancestors in the pedigree file. The number of equivalent generations for each animal (hereinafter called generations) was calculated by the equation: *g* = ∑(1/2)^*n*^, where *g* is the equivalent generation number and *n* is the number of generations separating the individual from each known ancestor. The method used is similar to the equation described by Welsh et al. (2010).

A summary of the pedigree analysis of the Brangus bulls used is shown in the Supplementary Material (Supplementary Figure 1). The index of pedigree completeness (PCI) was 0.94 (±0.143 SD), computed following the MacCluer et al. (1983) algorithm. PCI is the harmonic mean of the pedigree completenesses of the parents, summarizing the proportion of known ancestors in each ascending generation. Pedigree inbreeding of 0.04 (±0.035) was found for the breed. The average pedigree relationship was 0.086 (±0.081), and only 1.69% of the pairs had a pedigree relationship higher than 0.3.

### Filtering and Quality Control of Genomic Data

Markers with a call rate lower than 95% or not physically mapped to the bovine genome assembly UMD3.1 were removed from the analyses. The remaining genotypes were 698,282 SNP markers on the autosomes and 38,581 SNP on the sex chromosomes (37,538 in *X* and 1,043 in *Y*). Markers with minor allele frequency lower than 1% were removed. One Brangus sample with a call rate lower than 90% was removed.

### Runs of Homozygosity and Selection Signatures

The runs of homozygosity (ROH) analyses were conducted in SNP and Variation Suite^®^ v8.7 (Golden Helix, Inc., Bozeman, MT,^2^). The parameters were set to a minimum run length equal to 1000 kb with minimum of 70 SNP, allowing runs to contain up to two heterozygotes and five missing genotypes with a maximum gap equal to 50 kb and minimal SNP density of 1 SNP per 50 kb.

The minimal number of SNP to constitute a ROH (*l*) was determined by the same method used by Purfield et al. (2012) and determined by Lencz et al. (2007):

(1) $$l=\frac{\log_{e}\frac{\alpha }{ns.ni}}{\log_{e}\left(1-het\right)},$$

where *ns* is the number of SNPs per individual, *ni* is the number of individuals, α is the percentage of false positive ROH (set to 0.01 in this study), and *het* is the mean SNP heterozygosity across all SNP.

The incidence of common ROH was transformed to each breed’s frequency, dividing by the number of animals of each breed in the analysis. Normality tests were performed, and the frequency threshold defining the top 1% of the observations for each breed was determined. The homozygous regions above the frequency threshold of each breed (38% for Angus, 25.4% for Brahman, and 25.9% for Brangus) were considered as selected regions.

According to the length of the ROH, it is possible to estimate the number of generations traced back to the common ancestor, which generates the homozygosity in that region. We classified the ROH into 4 classes (1 = more than 10 generations, 2 = between 5 and 10, 3 = between 3 and 5, and 4 = less than 3 generations) using the equation proposed by Curik et al. (2014): *E*(*L*_IBD−H_|*gcA*) = 100/(2*gcA*), where *E*(L_IBD–H_ | gcA) is the expected length of an identical by descendent (IBD) haplotype (in centiMorgans – cM), and *gcA* is the number of generations from the common ancestor. The conversion from the recombination rate metric to physical distance (from cM to Mb) was performed using the average of the results of Arias et al. (2009) and Weng et al. (2014). Based on the Curik et al. (2014) equation, for example, an ROH longer than 13 Mb has most likely originated from a common ancestor less than three generations ago.

A genomic inbreeding coefficient based on ROH (*F*_ROH_) was calculated on each animal according to McQuillan et al. (2008) with the equation

(2) $$F_{\text{ROH}}=\frac{\sum_{j=1}^{n}L_{\text{ROH}j}}{L_{\text{total}}},$$

where *L*_ROHj_ is the length of ROH_j_, and *L*_total_ is the total size of the autosomes (using the estimated value in the Btau5.0.1 genome assembly of 2,522,199,562 bp). For each animal, *F*_ROH_ was calculated based on each of the four classes explained before and for each chromosome using the total size of each chromosome as *L*_total_ (following the chromosome size estimated by the Btau5.0.1 genome assembly).

### Chromosome Painting

We used the copying model, implemented in ChromoPainter (Lawson et al., 2012), to estimate the ancestry of regions across each genomic region. This copying model relates the patterns of linkage disequilibrium (LD) across chromosomes to the underlying recombination process. The method uses a hidden Markov model to reconstruct a sampled haplotype. To reinforce chromosome-painting results, we ran Fst analyses (Weir and Cockerham, 1984) for each region comparing the pairs (Angus vs. Brangus and Brahman vs Brangus). The function–fst in the plink1.9 software^3^ was used.

We used the founder breeds, Angus and Brahman, as haplotype donors to the Brangus haplotypes. The ChromoPainter analyses were performed twice (allowing or not allowing self-copying) using the linked model. The recombination files were created using the Perl scripts provided on the ChromoPainter website^4^. Beagle3.3 (Browning and Browning, 2007) was used to phase the genotypes (using 20 iterations).

### Simulation Model

We performed a population genetics simulation using the online tool^5^. The initial parameters were set to an initial allele frequency of 62.5% (representing the Angus allele in the first generation of Brangus); 10 generations; effective population size of 100; no selection, mutation, migration, and inbreeding (similar to a neutral model). We performed 50 simulations for each generation. The raw data were used to calculate the summary statistics (mean and standard deviation) and to determine the expected lower and upper value (within 99% of the Gaussian distribution) of the expected founder composition for each locus. These lower and upper values were applied as a threshold in the visualization of chromosome painting results to identify regions with significant enrichment of alleles coming from one of the founders.

### Identification of Genes and QTL in Selective Signatures

Genes in the selected regions (ROH islands) were identified in the Golden Helix GenomeBrowse^®^ visualization tool v2.1 (Golden Helix, Inc, Bozeman, MT,^6^). The genes were identified based on the NCBI *Bos taurus* annotation release 105 and Btau5.0.1 genome assembly. The genes list obtained was submitted in the NetworkAnalyst online tool^7^, aiming to characterize the biological process of these genes through the Enrichment Network tool using the PANTHER database. Thereafter, a search in the literature and in the Cattle QTL database (available online at^8^) was executed to identify traits related to genes located in each significant genomic region.

## Results

**FIGURE 1:**
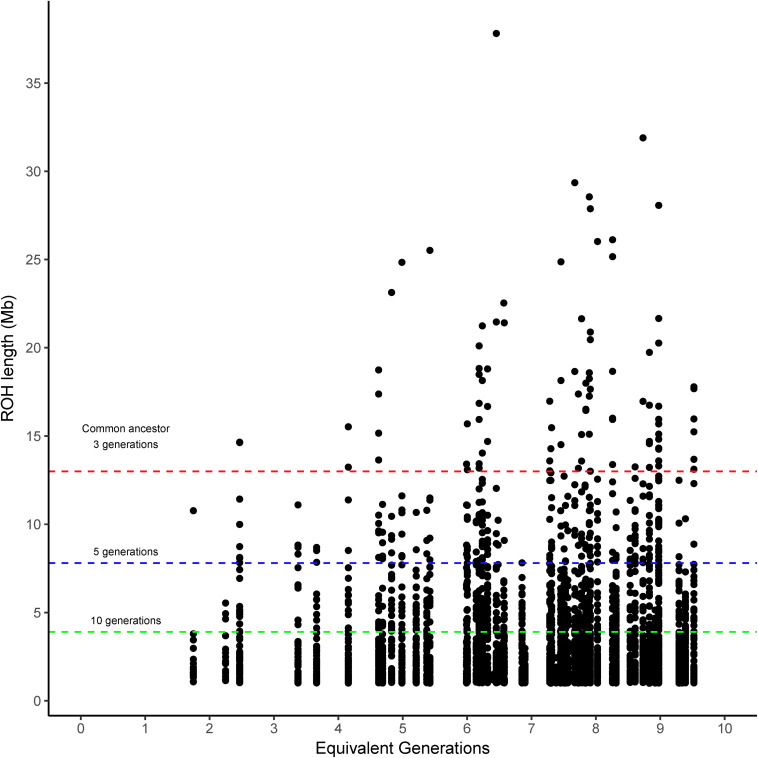
Runs of homozygosity (ROH) length observed in Brangus cattle according to the equivalent generation number of each animal. Dashed lines indicate the length threshold for ROH that relates to a common ancestor at 3, 5, and 10 prior generations (red, blue, and green, respectively) following the equation proposed by Curik et al. (2014).

The runs of homozygosity (ROH) were categorized into four classes according to the expected number of prior generations to a common ancestor (>10, >5, >3, and <3 generations). The ROH classified as coming from a common ancestor within the previous 3 generations (>13 Mb) was found in Brangus between the 4th and 5th generations, and the incidence increased thereafter for most chromosomes (Figure 1). That said, 54.2% of Brangus animals had long ROH (>13 Mb) indicative of recent inbreeding. However, chromosomes 17, 23, 26, and 28 did not have any ROH in this length range (Supplementary Figure 1).

**FIGURE 2:**
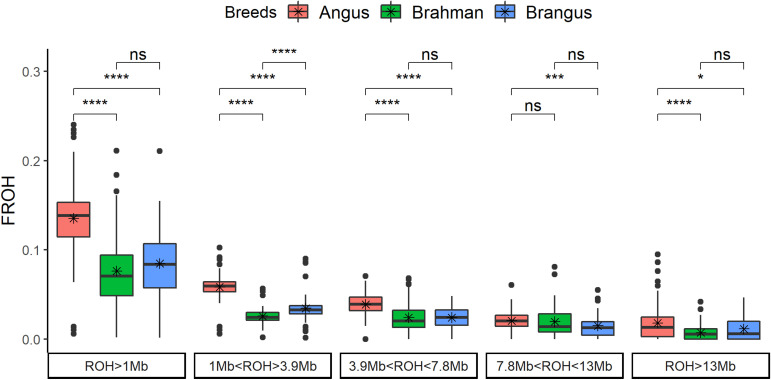
Genomic inbreeding based on runs of homozygosity (*F*_ROH_) by breed and by ROH length classes. The *t*-test comparison results are shown in the top (ns: not significant; *p < 0.05; **p < 0.01; ***p < 0.001; ****p < 0.0001).

The genomic inbreeding coefficient based on ROH (*F*_ROH_) was significantly (*p* < 0.0001) higher for Angus cattle compared to Brahman and/or Brangus (Figure 2). Brangus had lower *F*_ROH_ than Angus for all classes. Brahman and Brangus cattle had the same *F*_ROH_ for the ROH coming from a common ancestor tracing through 10 generations (all classes with ROH > 3.9 Mb), which was not expected and suggests a high effective population size for Brahman. For ROH coming from more than 10 previous generations (ROH < 3.9 Mb), Brangus cattle had higher *F*_ROH_ than Brahman cattle.

Pedigree inbreeding had a positive and significant relationship with *F*_ROH_, and a similar pattern was observed in all the classes of ROH length (Supplementary Figure 2). Brangus cattle had 8.5 ± 3.97% of genomic inbreeding and 3.9 ± 3.41% of pedigree inbreeding. Animals with no inbreeding at pedigree had close to 6% of genomic inbreeding. Averaged across chromosomes, the rate of *F*_ROH_ increased ≈1% per generation in Brangus (*F*_ROH_ = 0.0196 + 0.0097^∗^generation, $R_{adj}^{2}$ = 0.19, *p*-value = 0.0004). The increase in *F*_ROH_ was not observed for all chromosomes; only chromosomes 4, 10, 13, 15, 23, 26, and 29 had a positive *F*_ROH_ slope with generation number (Supplementary Figure 3). All the aforementioned chromosomes, except for 13, had a high proportion of Angus composition.

**FIGURE 3:**
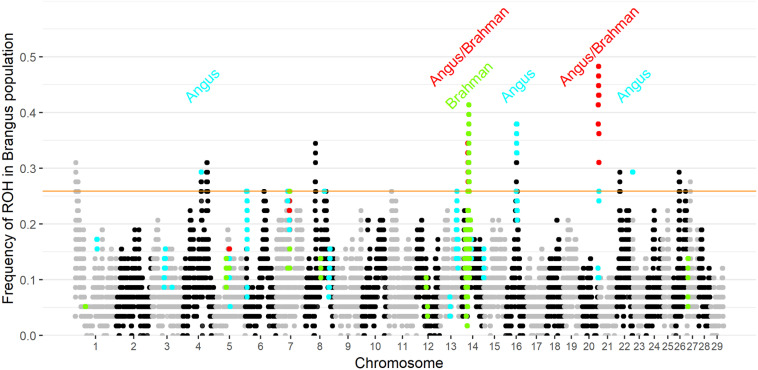
Frequency of each SNP in a run of homozygosity (ROH) in Brangus population according to the chromosome and position. The orange horizontal line indicates the 1% threshold to classify the SNP to be in an ROH island. Highlighted points indicate SNP above the 1% threshold in the founder breeds (blue for Angus, green for Brahman, and red in both founder breeds).

Ten genomic regions had ROH with frequency higher than 25.9% in Brangus (the top 1% of ROH frequency). Two of the 10 regions were found to be ROH islands for both founder breeds, and three ROH islands were observed in Angus (Figure 3). ROH above a 1% threshold were identified in 10 and 21 regions for Brahman and Angus, respectively (Supplementary Figure 4).

**FIGURE 4:**
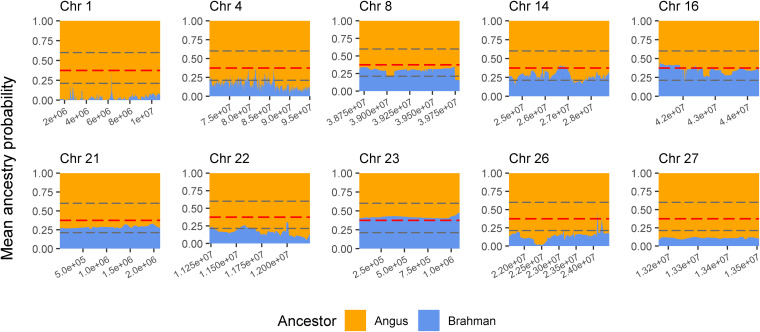
Probability of ancestry for regions in chromosomes (Chr) 1, 4, 8, 14, 16, 21, 22, 23, 26, and 27 identified as a selection signature (ROH island) in Brangus animals (above the top 1% threshold, 25.9% for Brangus). The plots show the average probability of ancestry according to the position in the region calculated from chromosome painting results. Horizontal dashed line in gray represent the expected maximum (top 1%) and minimum (bottom 1%) threshold for Brahman ancestry according to simulated data.

The genes and known QTLs within homozygous Brangus regions are shown in Table 1. The main biological process observed in gene network enrichment analysis from these homozygous regions were bile acid metabolic process, fatty acid beta oxidation, pentose phosphate shunt, neuron synaptic transmission, protein folding, regulation of cell cycle, cholesterol metabolic process, and unsaturated fatty acid biosynthesis. The main traits observed in QTL analysis of these regions were body weight, milk fat, calving ease, milk production, milk protein, body weight at birth, and fat thickness at the 12th rib (Supplementary Figure 5). The breed of origin of these regions was investigated using chromosome painting (Figure 4). *F*_ST_ results show Brangus had a closer relationship with Angus than with Brahman in these ROH regions (Table 1). The haplotypes in the regions of chromosomes 1, 4, 22, 26, and 27 appear to have originated from Angus. The regions in chromosomes 8, 14, 16, 21, and 23 have a mixture of Angus and Brahman origin, falling within the range of expected ancestry based on the whole genome.

**TABLE 1:** Homozygous regions observed in Brangus animals and the identification of genes underlying QTL in each region.

Chr^a^	Start (Mb)	End (Mb)	Length (Mb)	nSNPs^b^	Angus^c^	F_ST_^d^		nGenes^e^	nQTLs^f^	nTraits^g^	Genes associated with traits^h^
						Angus	Brahman				
1	1.56	10.78	9.22	2763	96.5%	0.057	0.277	23	50	33	POLLED locus, ADAMTS5 (milking speed), IFNAR1 (fat thickness at the 12th rib), CCT8 (conception rate, net merit)
4	70.02	71.46	1.44	514	81.8%	0.014	0.074	16	4	4	
4	91.47	95.01	25.00	1060	90.4%	0.067	0.406	76	33	31	Leptin (feed intake and energy balance), AHCYL2 (Longissimus muscle area)
8	38.70	39.80	1.10	225	70.6%	0.120	0.489	30	14	9	
14	24.42	28.79	4.37	1331	72.4%	0.069	0.214	38	280	34	XKR4 (heifer pregnancy, prolactin level, scrotal circumference, subcutaneous rump fat thickness), PLAG1 (average daily gain, body weight, carcass weight, intramuscular fat, longissimus muscle area, marbling score, scrotal circumference, stature), CHCHD7 (stature), SDR16C5 (fat color in carcass, insulin-like growth factor 1 level, milk fat percentage, scrotal circumference, beta-carotene concentration in fat), SDR16C6 (insulin-like growth factor 1 level, scrotal circumference, stature), FAM110B (carcass weight, insulin-like growth factor 1 level), SDCBP (carcass weight), TOX (carcass weight, insulin-like growth factor 1 level), CA8 (insulin-like growth factor 1 level, milk protein yield), RAB2A (carcass weight), CHD7 (insulin-like growth factor 1 level)
16	41.24	44.36	3.12	711	65.3%	0.146	0.44	75	562	46	
21	0	2.13	2.13	155	71.7%	0.23	0.613	27	78	9	
22	11.24	12.22	0.99	243	83.6%	0.181	0.281	24	23	20	
23	0	1.09	1.09	167	58.4%	0.058	0.092	1	28	23	KHDRBS2 (calving ease, daughter pregnancy rate, foot angle, milk fat percentage, milk fat yield, length of productive life, milk protein percentage, somatic cell score, stillbirth, strength)
26	21.56	24.46	2.90	672	86.3%	0.107	0.306	76	315	57	BTRC (milk c14 index, milk myristoleic acid content), SUFU (milk c14 index, milk myristoleic acid content, udder structure), CNNM2 (milk c14 index, milk myristoleic acid content, stearic acid content), INA (myristoleic acid content), NT5C2 (milk c14 index)
27	13.17	13.51	0.34	92	88.9%	0.145	0.319	8	25	12	

*Genes were identified on the NCBI Bos taurus Annotation Release 105 and Btau5.0.1 genome assembly. ^a^Chromosome. ^b^Number of markers (SNP) inside the region in homozygosity. ^c^Probability of the region coming from Angus according to chromosome painting results. ^d^F_ST_: Fixation index (Weir and Cockerham, 1984). ^e^Number of genes inside the region. ^f^Number of QTLs identified in Cattle QTL database. ^g^Number of traits associated with the QTLs. ^h^Genes in the region that area associated with a trait (traits of each gene between parenthesis).*

## Discussion

Overall, Brangus had 63% of the inbreeding level of Angus, based on runs of homozygosity (ROH) (Figure 2). Brangus had higher inbreeding than Brahman only in the shortest category of ROH (<3.9 Mb), which suggests the number of generations after crossbreeding was not sufficient to break down short ROH. Brangus had a higher Angus proportion (70.4%) than expected (62.5%) in the whole genome (Paim et al., 2020). The high Angus proportion might be related with the initial crossbreeding to develop the composite associated with genetic drift and selection for specific traits (Paim et al., 2020). Therefore, the higher Angus proportion may be linked to this excess of short ROH in Brangus compared to Brahman. Moreover, inbreeding across chromosomes was not equal; this may suggest that new levels of homozygosity are starting to form as a function of selection pressure and the use of sires that are deemed superior to their contemporaries.

The length of ROH agree with the generation criteria of Curik et al. (2014). The ROH coming from a common ancestor within 3 prior generations (>13 Mb) appeared between the 4th and 5th generation and increased afterward, suggesting inbreeding has started to accumulate in this relatively new breed.

The inbreeding level increased approximately 1% per generation corresponding to an effective population size (Ne) of 51.55 (Ne = 1/2Δ*F*) (FAO, 2013). According to FAO Guidelines for *in vivo* conservation of animal genetic resources (FAO, 2013), the desired inbreeding rate per generation should not exceed 1% (equal to Ne = 50). A 1% increase in inbreeding was associated with decrease of −0.23% in yearling weight and −0.64% in body condition score in a tropical composite beef cattle (Reverter et al., 2017). Therefore, selection pressure and finite population size promotes increased inbreeding, suggesting that inbreeding management remains important for composite breeds.

Three selected regions in Brangus (chromosomes 4, 16, and 23) were identified as Angus selection signatures, and two regions on chromosomes 14 and 21 were identified as selection signatures in both founder breeds (Figure 3). Chromosome painting results showed that five of the 10 homozygous regions in Brangus were predominantly Angus in origin (probability >80%), and the other five regions were of mixed origin but always with Brahman contributing less than 50% (Figure 4).

The traits associated with the predominantly Angus regions identified in the Cattle QTL database were body condition, body weight, calving ease, birth weight, fat thickness at the 12th rib, and milk traits. For example, the region on chromosome 23 (0–1090080bp) with high ROH frequency in Angus and Brangus harbored the *KHDRBS2* gene, which was previously associated with calving ease (Cole et al., 2011).

One homozygous locus on chromosome 4 contained the *LEP* gene, which is associated with 96 traits in the Cattle QTL database. This gene is expressed in adipose tissue and codes for leptin, a hormone known to regulate feed intake and energy balance in mammals (Woronuk et al., 2012). This gene had been associated with marbling, fat thickness, rib eye area, and feed intake in several beef cattle breeds (Souza et al., 2010; Woronuk et al., 2012; Kononoff et al., 2017). Leptin is considered an extremely important gene for puberty onset (Williams et al., 2002). A high Angus contribution (90.4%) to this homozygous region was identified in Brangus (Table 1). Therefore, an allele coming from Angus was probably selected in Brangus.

Another homozygous region in chromosome 16 also was associated with first service conception in yearling Brangus heifers (Peters et al., 2013). *Bos indicus*–influenced heifers are known to have challenges achieving puberty early in life (Sartori et al., 2010; Fortes et al., 2012b). Therefore, high selection pressure in Brangus for early puberty since breed formation probably existed.

Another homozygous region (BTA 14) was previously identified as a QTL for weaning weight in Brangus (Weng et al., 2016). Cánovas et al. (2014) reported two genes on BTA14 at 24Mb associated with Brangus heifer fertility traits. This region harbors *PLAG1* and *XKR4* genes. The *XKR4* was associated with subcutaneous rump fat thickness, scrotal circumference, serum concentration of prolactin, and sexual precocity (Fortes et al., 2012a; Porto Neto et al., 2012; Bastin et al., 2014; Takada et al., 2018). *PLAG1* has been implicated in the regulation of stature and weight (Littlejohn et al., 2012; Pryce et al., 2012; Song et al., 2016). This gene was associated with yearling weight in Australian Tropical Composite breeds (Porto-Neto et al., 2014). The association studies of these genes used both taurine and indicine cattle, which confirms our observation of a selection signature in both founder breeds and a mixed origin of this region in Brangus.

The C allele of a putative functional mutation (rs109231213) near *PLAG1* significantly increased hip height, weight, net food intake, age at puberty in males and females and decreased concentration of IGF-I in blood and fat depth (Fortes et al., 2013). These authors reported that haplotypes carrying the C allele had the same surrounding 10 SNP haplotype in *B. taurus* and Brahman, probably because the C allele was introgressed into Brahman from *B. taurus* cattle. The region with reduced heterozygosity surrounding the C allele was small in *B. taurus* and in Angus in this study (1.7 Mb) but 21.6 Mb long in Brahmans, here as well as in Fortes et al. (2013). Therefore, this allele represents a mutation that has been selected almost to fixation in *B. taurus* and, likely, introduced into Brahman cattle during crossbreeding with taurine cattle when indicine cattle were introduced into the United States (Sanders, 1980; Fortes et al., 2013).

Selection for growth and growth-related traits, such as average daily gain, feed conversion, and body size, has been conducted to improve beef productivity in both taurine and indicine breeds in the United States for several decades (Willham, 1982). Therefore, it is likely that favorable alleles for growth in genes with large phenotypic effects have also increased in frequency in both and the distribution of allele frequencies at these QTL have become similar between both populations.

The high Angus contribution for the selected genomic regions in Brangus cattle could support the use of the Brangus data for genomic selection and QTL identification (fine mapping) for Angus. This reinforces previous simulation studies that a crossbred or an admixed population can be used as training data for genomic selection and can provide reasonably accurate estimates of genomic breeding values of purebred selection candidates (Toosi et al., 2010). Marker estimates obtained from crossbred populations can be used to select purebreds looking for crossbred performance (Ibanez-Escriche et al., 2009; Toosi et al., 2010; MacNeil et al., 2011; Zeng et al., 2013; Lopes et al., 2017). Moreover, the results highlight how selection criteria can shape the genetic makeup of the composite.

The genetic composition of a composite breed is dynamic and changes across generations (Paim et al., 2020). Here, the selected regions in Brangus were mainly from Angus. The core idea of developing a composite breed is to exploit heterosis and complementarity between the breeds and, in the Brangus example, explore combining the tropical adaptation of zebu cattle and high yield and meat quality of Angus. These results and those previously reported (Paim et al., 2020) suggest Brangus is moving toward traits where Angus excel due to the selection imposed by breeders. Yield and meat quality (marbling) are measured and genetic values are available in the association’s breed improvement program. The “tropical adaptation” traits, however, are not measured, and consequently, there is no genetic evaluation for their improvement. Therefore, it is important to develop and apply methods of measuring tropical adaptation and selecting for it; otherwise, this beneficial attribute of Brangus could be lost in future Brangus generations.

## Conclusion

The majority of selection signatures in Brangus cattle came from Angus, which can be related to the traits of interest for genetic improvement and selection. These results demonstrate how quickly selection and drift can shift the genetic architecture of a population. Genomic inbreeding was found to be increasing in the composite population with advancing generations. Therefore, breeders should be aware of the need to manage inbreeding in this population. Moreover, composite cattle breeders need to be aware that selection for a set of specific traits that favor one of the progenitor breeds over the other can and will alter the original breed proportions and which, over the long term, may decrease the utility of the composite.

## Data Availability Statement

The datasets generated for this study can be found in the all genotypic data used for this study are available in the website of The Animal-Genetic Resources Information Network (Animal-GRIN) (https://nrrc.ars.usda.gov/A-GRIN).

## Ethics Statement

Ethical review and approval was not required for the animal study because no samples were collected for this study; rather they were collected as part of other studies or program activities not associated with this study.

## Author Contributions

TP analyzed the data, interpreted the results, and wrote the manuscript. EH analyzed the data, discussed the results, and revised the text. CW maintained the datasets and analyzed the data. MT discussed the results and revised the text. LK was responsible for data curation, discussion of the results, and revision of the text. SP designed the study, discussed results, and revised the text. CM designed the study and revised the text. HB coordinated the study, discussed and interpreted the results, and wrote the manuscript. All authors read and approved the final manuscript.

## Conflict of Interest

The authors declare that the research was conducted in the absence of any commercial or financial relationships that could be construed as a potential conflict of interest.

## Footnotes

1 https://gobrangus.com/

2 www.goldenhelix.com

3 www.cog-genomics.org/plink/1.9/

4 http://www.paintmychromosomes.com/

5 http://popgensimulator.pitt.edu/graphs/allele

6 www.goldenhelix.com

7 https://www.networkanalyst.ca/

8 http://www.animalgenome.org
