# Supplementary material for: Genomic Breed Composition of Selection Signatures in Brangus Beef Cattle
Source: Front Genet. 2020 Jul 10;11:710. doi: 10.3389/fgene.2020.00710 (PMC7365941; doi:10.3389/fgene.2020.00710)
Supplement: Supplementary file 1 [file Table_1.DOCX]

Supplementary Material





**Supplementary Figure 1.** Pedigree information of the Brangus bulls used in the study extract with the optiSel package (Wellmann, 2017) in R 3.4.2 software (R Core Team, 2017).

**
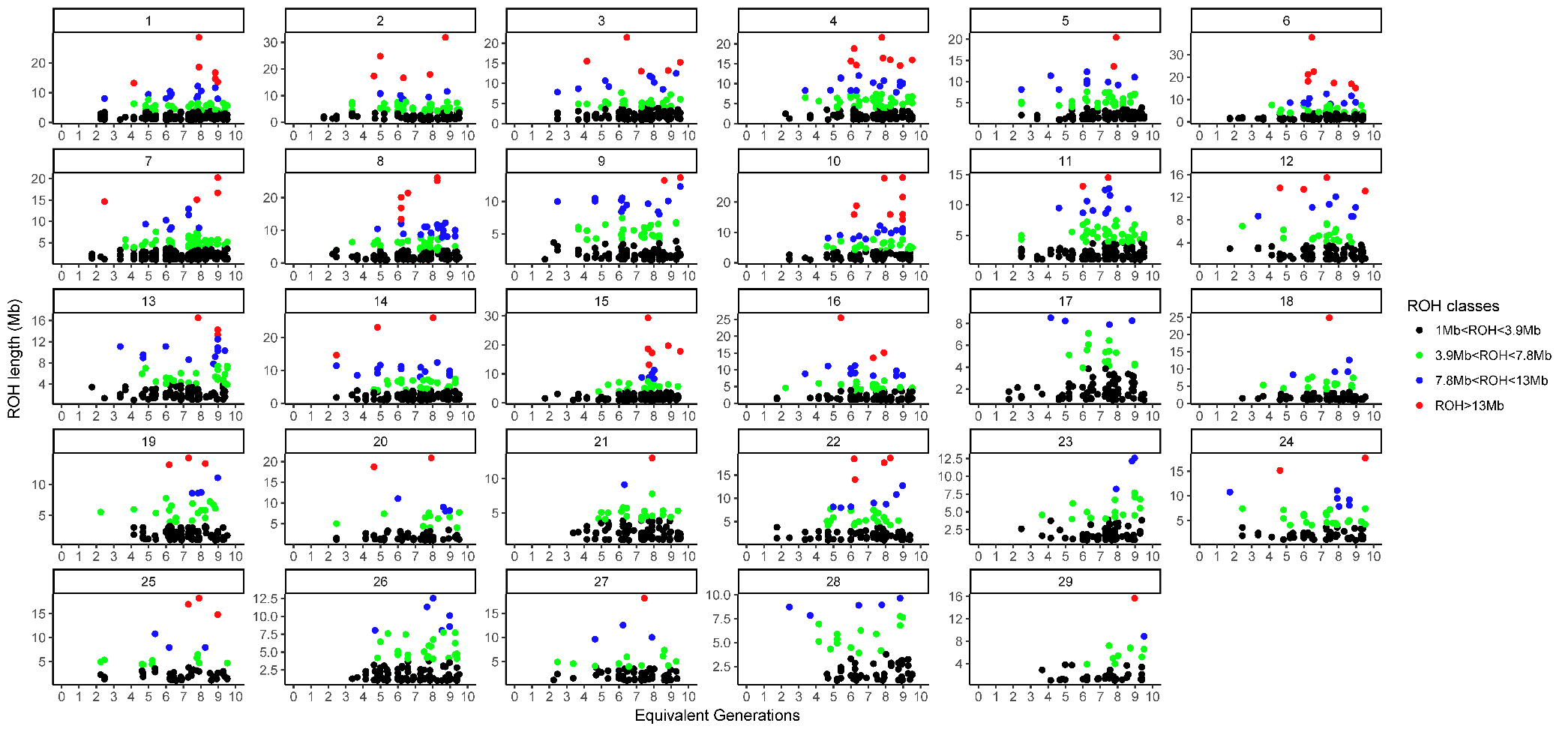
**

**Supplementary Figure 1.** Runs of homozygosity (ROH) length observed in each autosome (1 to 29) in Brangus cattle according to the equivalent generation number of each animal. For each chromosome (1-29), the points were colored according to the length threshold for ROH that relates to a common ancestor at 3, 5 and 10 prior generations (red, blue and green, respectively) following equation of Curik et al. (2014).

**
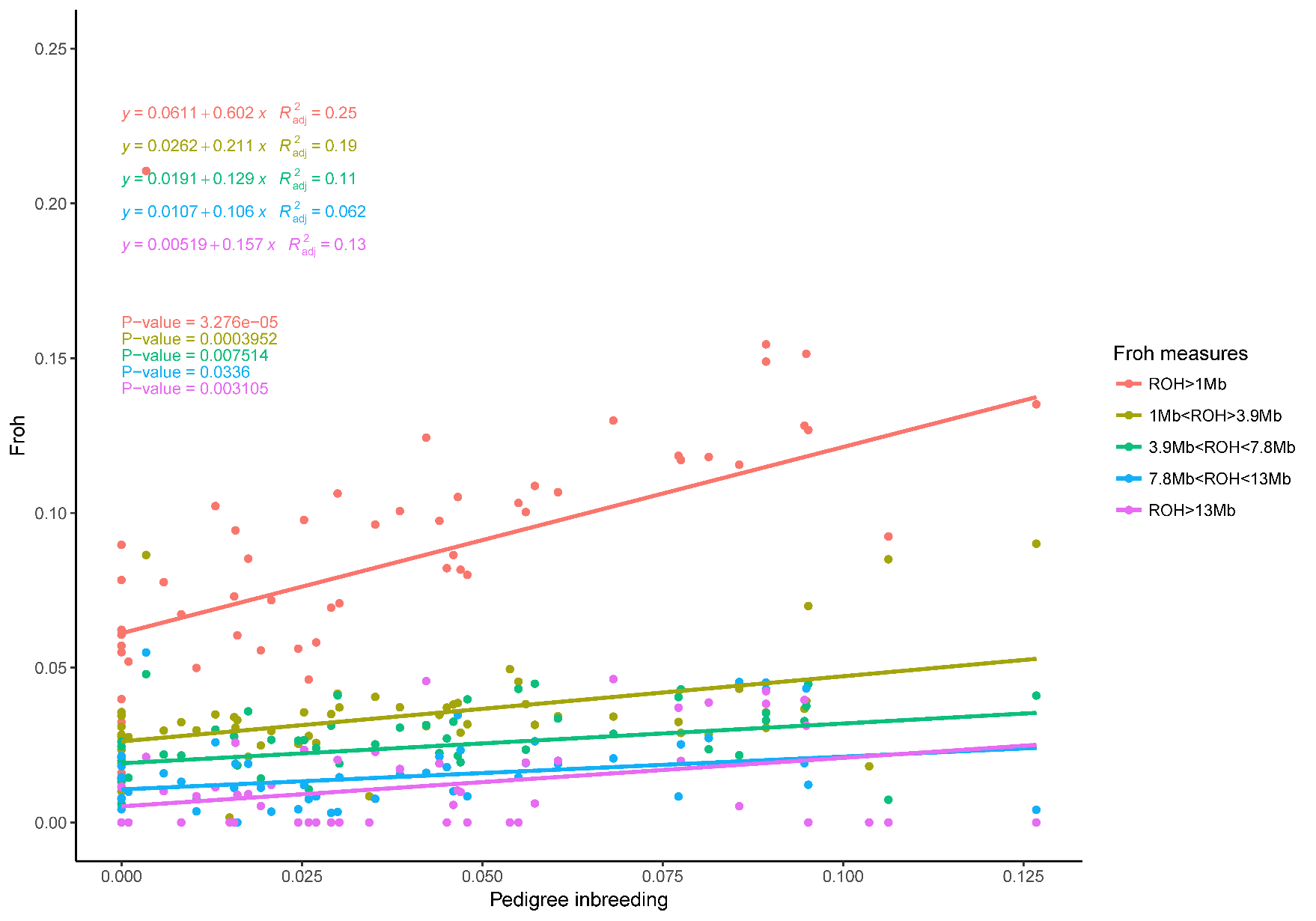
**

**Supplementary Figure 2.** Linear regression analyses between pedigree inbreeding and genomic inbreeding based on runs of homozygosity (F_ROH_) using autosomes SNP. Runs of homozygosity (ROH) classified by the length threshold that relates to a common ancestor at 3, 5 and 10 prior generations (pink, blue and green, respectively) following equation proposed by Curik et al. (2014).


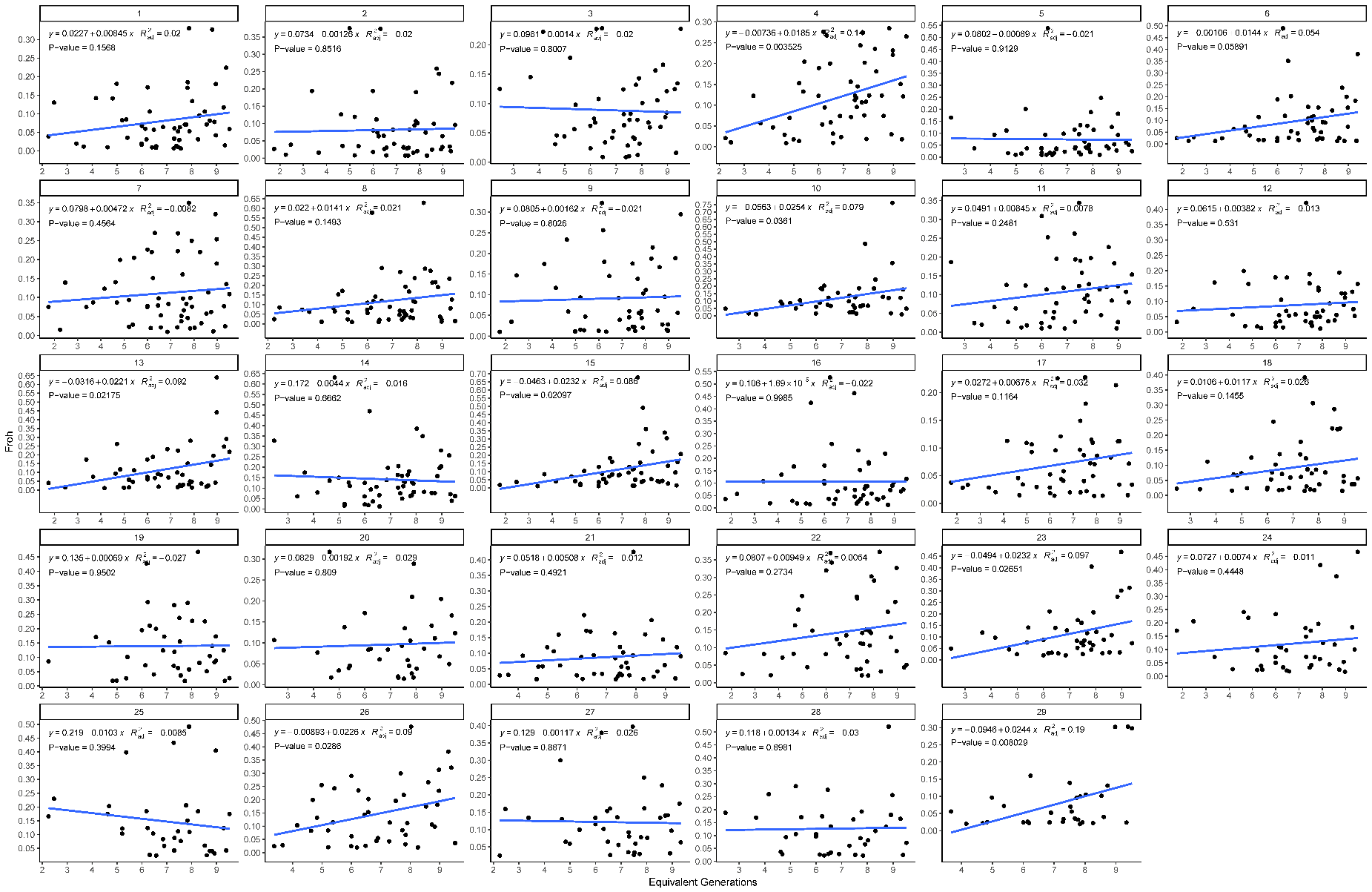


**Supplementary Figure 3**. Linear regression analyses between number of equivalent generations and genomic inbreeding based on runs of homozygosity (FROH) for each autosome in Brangus cattle.

**
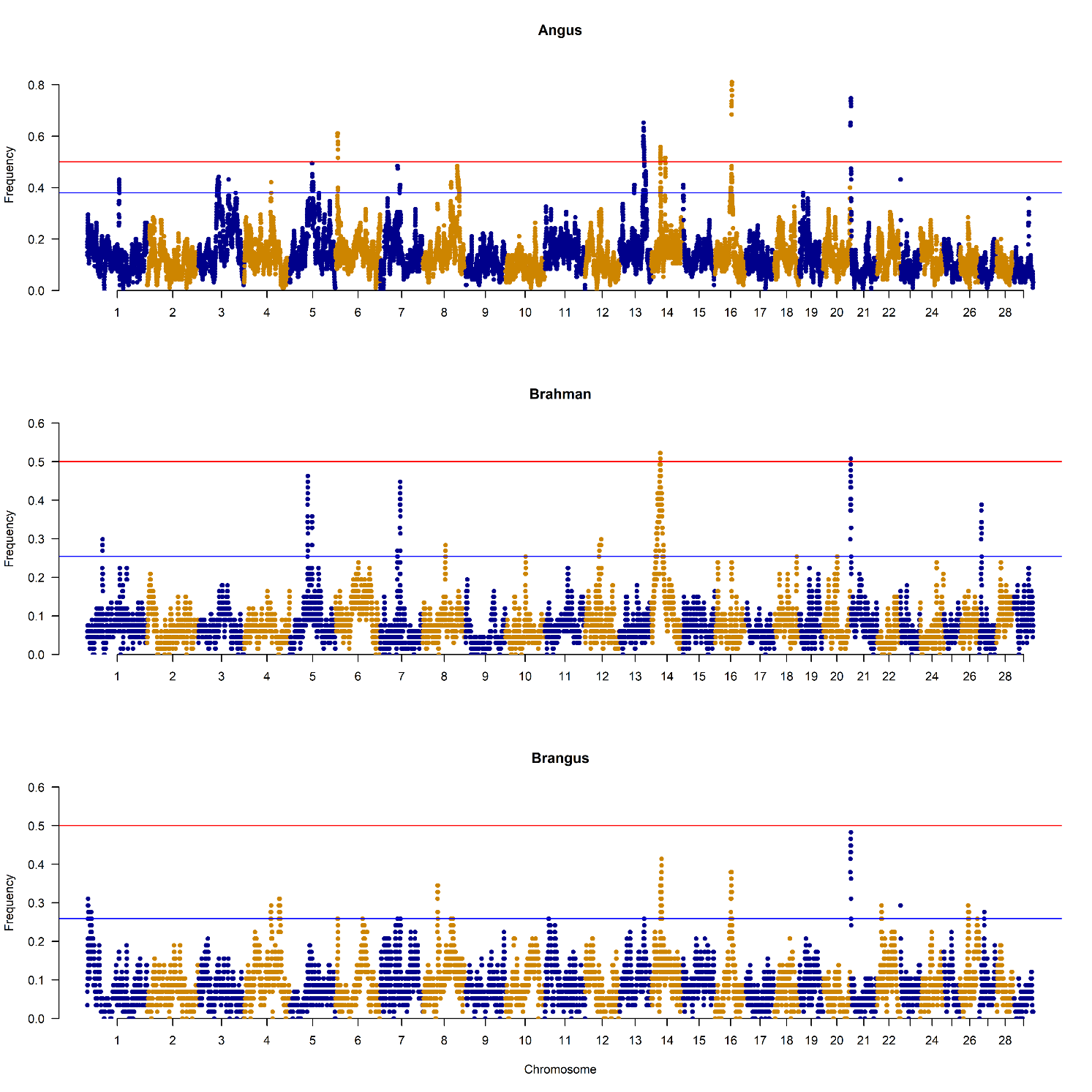
**

**Supplementary Figure 4.** Frequency of each SNP in a run of homozygosity (ROH) in each breed (Angus, Brahman and Brangus) according position within each chromosome. The blue horizontal line signifies the 1% threshold to classify the SNP as an ROH island. The red horizontal line signifies the ROH frequency equal to 50% of the population (SNPs above this line are in a highly homozygous region in the population).


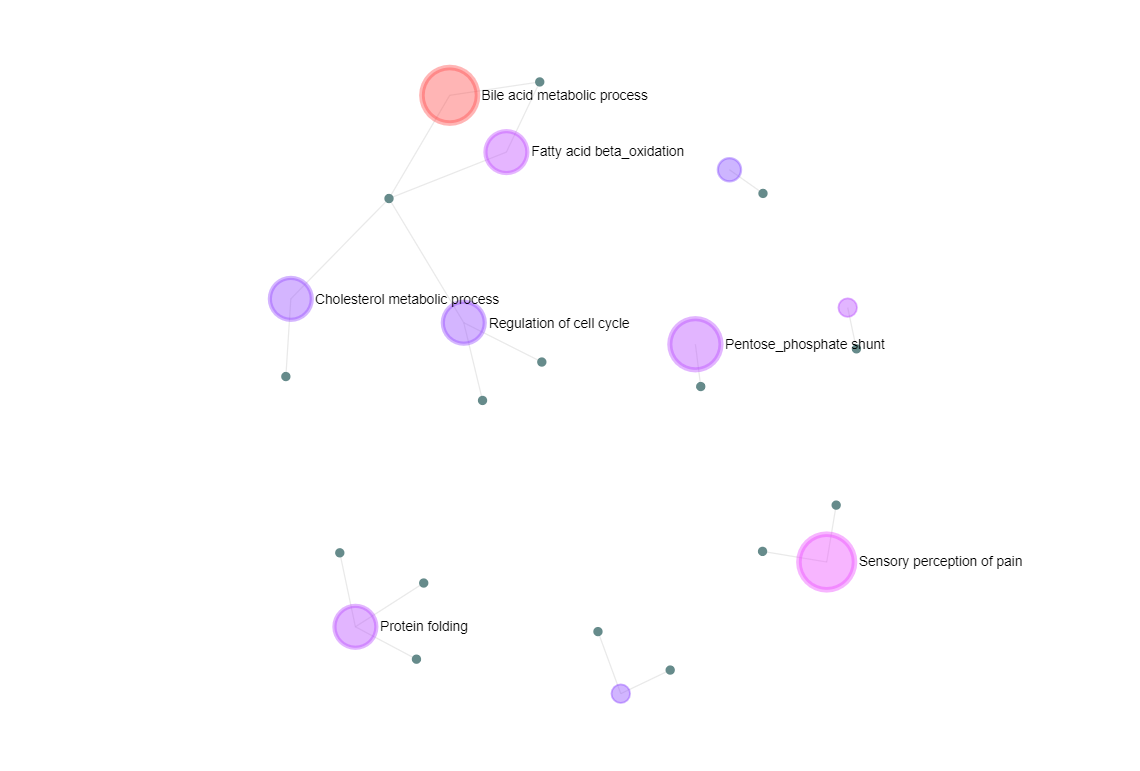

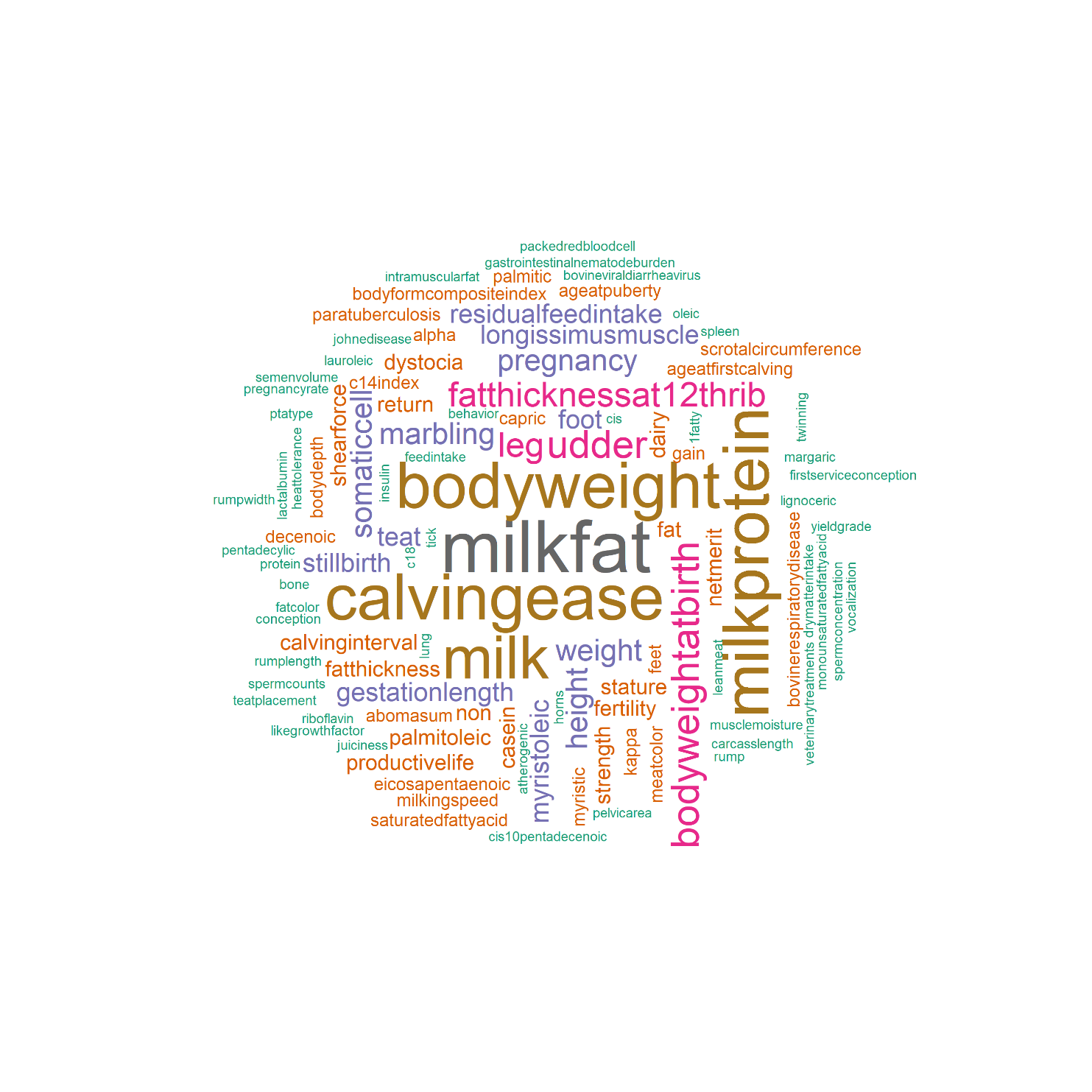


B

A

**Supplementary Figure 5.** Biological process (A) of the genes based on gene network analysis and word cloud (B) of the traits in known cattle QTLs in these ROH regions.
